# The rising challenge of *Candida auris*: insights into its transmission, drug resistance, and infection control strategies

**DOI:** 10.3389/fmicb.2025.1694108

**Published:** 2025-11-11

**Authors:** Weiming Zhang, Xiaoli Cao, Chang Liu, Shuo Gao

**Affiliations:** Department of Clinical Laboratory, Nanjing Drum Tower Hospital, Affiliated Hospital of Medical School, Nanjing University, Nanjing, China

**Keywords:** *Candida auris*, epidemiology, transmission routes, resistance, outbreak, infection prevention and control, prevention strategies

## Abstract

*Candida auris* (also known as *Candidozyma auris*) is a newly emerged pathogenic fungus that has garnered widespread attention globally, particularly in healthcare settings. Its rapid transmission, association high pathogenicity, and resistance to multiple antifungal agents have made it a significant public health challenge. *C. auris* commonly causes invasive infections in immunocompromised patients, and its resistance to various antifungal drugs complicates treatment strategies. This review summarizes the epidemiological characteristics, transmission routes, risk factors, and resistance mechanisms of *C. auris*, with a focus on its hospital transmission dynamics and environmental persistence. Additionally, we discuss current control and prevention strategies, including early detection, infection control measures, and the rational use of antifungal agents. Finally, the article looks ahead to future research directions, particularly the potential for vaccine development and immunotherapy, aiming to provide scientific insights for optimizing clinical diagnosis and treatment strategies.

## 1 Introduction

*Candida auris* (also known as *Candidozyma auris*) is an emerging pathogenic fungus that has garnered widespread attention in recent years. It was first isolated in 2009 from a patient with an ear infection in Japan (Satoh et al., 2009). Since its initial report, *C. auris* has rapidly spread globally, causing outbreaks of hospital-acquired infections in multiple countries and regions (Du et al., 2022; Mishra et al., 2023; Sanyaolu et al., 2022; Suphavilai et al., 2024). Compared to traditional *Candida* species, *C. auris* possesses several unique characteristics, including high drug resistance, rapid transmission, and the ability to cause widespread outbreaks, posing a particularly severe threat to immunocompromised or critically ill patients, who experience high mortality rates. This mortality is often attributable to a combination of factors including multidrug resistance, delays in diagnosis, and the vulnerable underlying conditions of the affected patients (Muñoz et al., 2018; Horton et al., 2023; Santana et al., 2023; Lockhart et al., 2023; Shastri et al., 2020; Das et al., 2018). The significant clinical challenge posed by *C. auris* stems from a combination of its multidrug resistance, prolonged environmental persistence, and high transmissibility in healthcare settings, rather than from an exceptionally high level of intrinsic virulence alone (Choudhury et al., 2025; Yune et al., 2023; Patterson et al., 2021). Its widespread transmission in hospitals and long-term care facilities has posed a significant public health challenge, especially in the context of the increasing ineffectiveness of antifungal drugs, making its resistance a major concern.

Currently, the complex epidemiological characteristics and transmission mechanisms of *C. auris* make it a focal point of global public health research (Mishra et al., 2023). Unlike traditional *Candida* species, *C. auris* not only spreads among infected patients but can also be transmitted through medical devices, environmental contamination, and cross-contamination among healthcare workers, quickly establishing transmission chains within hospital settings (Santana et al., 2023). This high transmissibility, coupled with its multidrug resistance, has made *C. auris* one of the most concerning pathogens in hospital-acquired infections. In recent years, *C. auris* has caused large-scale outbreaks in several countries and regions, particularly in intensive care units (ICUs) and other high-risk areas, where the infection rate is notably severe (Du et al., 2022; Suphavilai et al., 2024; Politi et al., 2024).

Although significant progress has been made in understanding *C. auris*, particularly regarding its resistance mechanisms and clinical manifestations, key issues such as its transmission routes, epidemiological characteristics, and prevention strategies remain crucial areas of ongoing research (Ganeshkumar et al., 2024). This review aims to provide a critical synthesis of the current knowledge on *C. auris*. A key contribution is the presentation of an integrative transmission model that links reservoirs, vectors, and evidence-based interventions in a single framework (see Table 1). This model, along with a detailed analysis of recent advances in resistance mechanisms, aims to offer a unique perspective for understanding and controlling this pathogen.

**Table 1:** Evidence-based strategies to break the transmission cycle of *Candida auris* in healthcare settings.

Transmission route /component	Key characteristics and persistence	Recommended control measures to break the route	Strength of evidence
Colonized/Infected Patient	Primary reservoir. Asymptomatic colonization is common and precedes infection.	1. Active surveillance screening (e.g., axilla/groin swabs) of high-risk patients and epidemiologically linked contacts. 2. Implementation of Contact Precautions (gowns, gloves) for all interactions with colonized or infected patients. 3. Patient isolation or cohorting in a single room or dedicated ward area.	Strong Observational [Supported by multiple outbreak control studies (Worth et al., 2020; Yang et al., 2025; Khanum et al., 2024)]
Healthcare Environment (Surfaces)	Can persist for weeks to months on surfaces. Demonstrates tolerance to some common hospital disinfectants.	1. Enhanced daily and terminal cleaning and disinfection using EPA-approved disinfectants with proven efficacy against *C. auris* (e.g., hydrogen peroxide-based cleaners, chlorine-based agents). 2. Prioritizing high-touch surfaces near the patient. 3. Monitoring cleaning efficacy (e.g., with fluorescent markers or ATP bioluminescence).	Experimental and Strong Observational [Laboratory data on disinfectant efficacy and observational outbreak data (Biswal et al., 2017b; Gülmez et al., 2022; Khanum et al., 2024)]
Medical Devices	Contaminated devices act as a direct portal of entry. Biofilm formation enhances resistance to cleaning and disinfection.	1. Use of dedicated disposable or single-patient-use non-critical medical equipment whenever possible. 2. Strict and meticulous protocols for cleaning and disinfection of shared medical devices between patients, adhering to manufacturer’s instructions. 3. Minimizing the use and duration of invasive devices when clinically safe to do so.	Strong Observational [Supported by epidemiological links during outbreaks and biofilm studies (Rudramurthy et al., 2017; Biswal et al., 2017b; Kauser et al., 2022; Khanum et al., 2024)]
Healthcare Workers’ Hands	HCWs’ hands are the primary vector for transient transmission between patients and the environment.	1. Strict adherence to hand hygiene using an alcohol-based hand rub (ABHR) or soap and water before and after patient contact and after touching the patient’s immediate environment. 2. Comprehensive education and compliance monitoring with feedback for HCWs.	Strong Observational [Based on fundamental infection control principles and consistent with outbreak control findings (Nevárez-Moorillón et al., 2025; Goulart et al., 2024; Biswal et al., 2017a)]

EPA, Environmental Protection Agency; ATP, adenosine triphosphate. Based on the current evidence, the transmission of *C. auris* within healthcare facilities can be conceptualized as a cycle involving four interconnected components. Breaking this cycle requires a bundled, targeted approach, as summarized in this table.

## 2 Search strategy and selection criteria

To ensure a comprehensive and reproducible overview of the current knowledge on *Candida auris*, a systematic literature search was conducted in PubMed, Web of Science, and Scopus for English studies published up to June 2024. The search strategy utilized key terms including “*Candida auris*” combined with relevant concepts such as epidemiology, transmission, outbreak, antifungal resistance, and infection control. After duplicate removal, articles were screened by title/abstract and then by full-text against predefined criteria: inclusion was limited to original research, case reports, and systematic reviews focused primarily on *C. auris* and providing data on epidemiology, transmission, resistance, or control strategies, while conference abstracts, non-peer-reviewed works, and unavailable full texts were excluded. Additional relevant publications were identified through manual screening of reference lists. Given the narrative nature of this review, evidence was synthesized qualitatively to thematically summarize current knowledge, highlight key findings and consensus, and identify evidence gaps and future research directions.

## 3 Epidemiological features of *Candida auris*

Since its discovery, *C. auris* has rapidly become a major pathogen of hospital-acquired infections globally. Its epidemiological characteristics indicate that *C. auris* tends to cause severe infections in immunocompromised patients and has high transmissibility and adaptability within hospital environments (Preda et al., 2024). Understanding the epidemiological features of *C. auris* is crucial for developing effective control and prevention strategies. The following are key epidemiological features of *C. auris*.

### 3.1 Global spread

**Figure 1:**
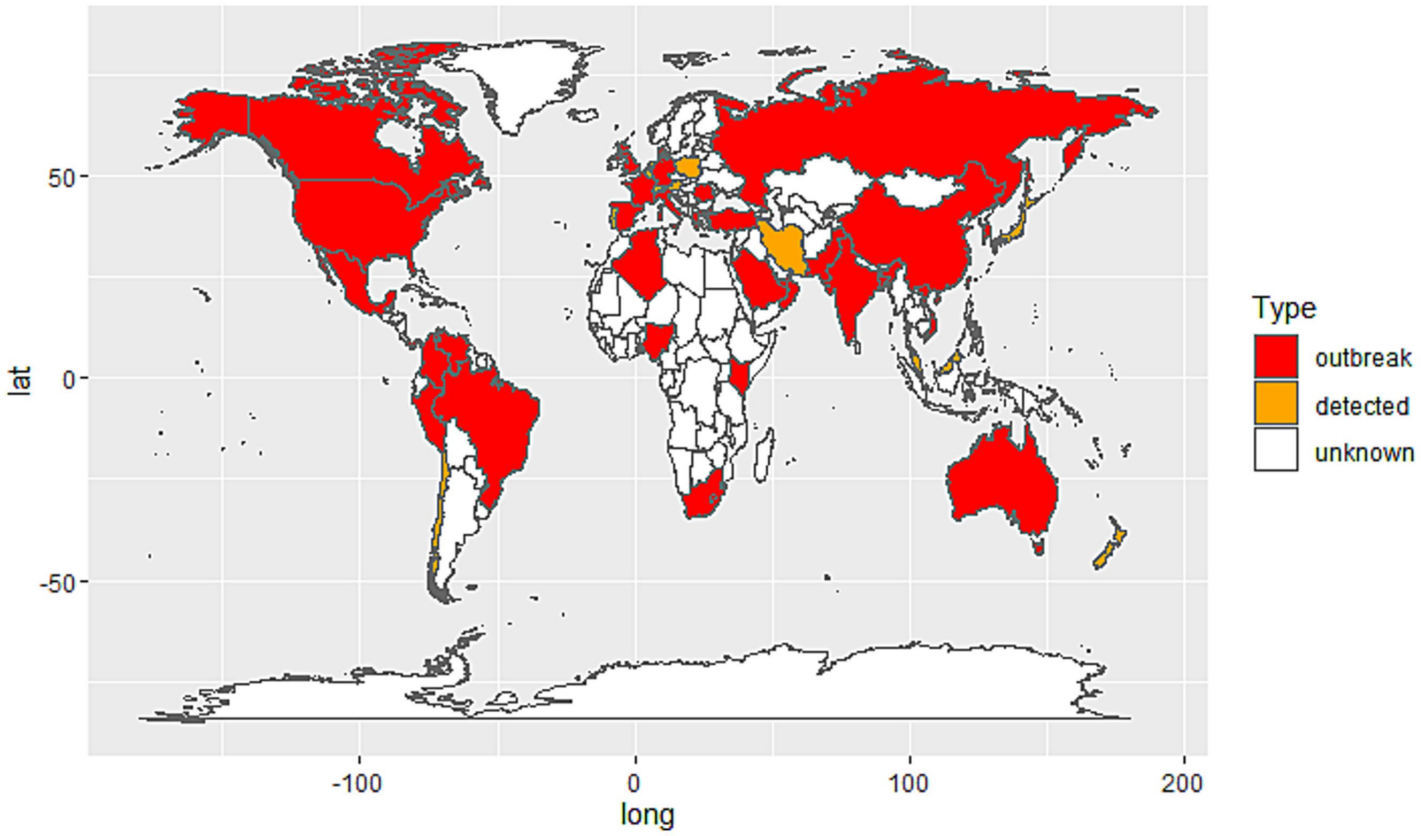
Global distribution of *Candida auris* cases by country. Red: Countries with documented *C. auris* outbreaks (confirmed local transmission or nosocomial clusters). Orange: Countries where *C. auris* has been detected but no outbreaks have been reported; White: Countries with no reported cases or insufficient data.

The spread of *C. auris* spans multiple countries and regions (Figure 1), with widespread hospital-acquired infection outbreaks, especially in hospital settings (Du et al., 2022; Suphavilai et al., 2024; Politi et al., 2024). The first case of *C. auris* was reported in Japan in 2009, followed by reports of cases in India, the United States, Europe, and several other countries (Ashraf et al., 2023; Zhu et al., 2020; Politi et al., 2024). According to monitoring data from the World Health Organization (WHO) and other public health agencies, *C. auris* has spread extensively in hospitals in countries such as the United States, India, Pakistan, Argentina, South Africa, and regions of Europe, becoming a global health threat (Politi et al., 2024; Ashraf et al., 2023; Zhu et al., 2020; Sasoni et al., 2022; Shuping et al., 2023; Farooqi et al., 2020).

The speed and extent of transmission within hospitals have exceeded the typical spread of many traditional fungi (Lass-Flörl et al., 2024). Several countries and regions have reported outbreaks of *C. auris*-caused hospital infections, particularly in intensive care units (ICUs), long-term care facilities, and among patients receiving immunosuppressive treatments, where the infection rate is notably higher (Politi et al., 2024; Ashraf et al., 2023; Long et al., 2024; Farooqi et al., 2020).

### 3.2 High-risk and susceptible groups

Infections caused by *C. auris* predominantly occur in patients with weakened immune systems or severe underlying conditions, especially the following high-risk groups: Immunosuppressed patients: Individuals undergoing chemotherapy, immunosuppressive drug treatments, organ transplantation, or suffering from AIDS or other immune system impairments (Wasylyshyn and Stoneman, 2024). Long-term hospitalized patients, particularly those in ICUs who have undergone multiple invasive procedures, are at an increased risk of *C. auris* infection (Du et al., 2020). Diabetic patients, due to their impaired immune function and hyperglycemic state, are more susceptible to infections by various pathogens, including *C. auris*. Neonates and the elderly, as their immune systems are relatively weak, are at increased risk, particularly premature infants and elderly patients. Patients receiving broad-spectrum antibiotics, as the widespread use of antibiotics disrupts the normal microbial flora, creating opportunities for colonization and spread of resistant pathogens like *C. auris* (Du et al., 2020; Munshi et al., 2024).

### 3.3 Clinical features of *C. auris* infections

Infections caused by *C. auris* are diverse, with common infection sites including the bloodstream, urinary tract, wounds, and ears. Because *C. auris* typically enters the body through invasive medical procedures, the types of infections it causes vary depending on the patient population (Wasylyshyn and Stoneman, 2024). Common types of infection include: Bloodstream infections (BSI): *C. auris*-caused bloodstream infections are among the deadliest infections in ICU patients, typically associated with high pathogenicity and mortality (Munshi et al., 2024). Urinary tract infections: especially in patients who have long-term use of urinary catheters, *C. auris* can cause urinary tract infections through the urethra (Griffith and Danziger, 2020). Ear infections: *C. auris* was initially isolated from ear infections, so it is more common in patients with ear-related conditions (Byun et al., 2023). Wound infections and infections in other organs: *C. auris* can also cause infections in sites such as the lungs, heart, and joints (Long et al., 2024). The clinical symptoms of *C. auris* infections are often subtle and can be confused with other types of fungal or bacterial infections, making early diagnosis and prompt treatment essential.

## 4 An integrative model of *C. auris* transmission in healthcare settings

The transmission of *Candida auris* within healthcare facilities is not a linear process but a dynamic cycle involving interconnected reservoirs and vectors (Yadav et al., 2021; Guan et al., 2025). Understanding this integrative model is crucial for developing effective intervention strategies. The cycle primarily involves four key components: colonized or infected patients, the healthcare environment, contaminated medical devices, and healthcare workers (HCWs).

### 4.1 The patient reservoir

Colonized and infected patients constitute the primary reservoir for *Candida auris* in healthcare settings, serving as the fundamental source from which transmission originates (Alvarado-Socarras et al., 2021). A critical aspect of its epidemiology is the high rate of asymptomatic skin colonization, particularly in body sites such as the axilla, groin, and nares (Politi et al., 2024; Piatti et al., 2022). This colonization is often persistent and can precede clinical infection by weeks or months (Das et al., 2018). Patients may unknowingly harbor the fungus on their skin or in the nares, functioning as continuous shedders that contaminate their immediate environment—including bed linens, bedside furniture, and medical equipment—through routine contact (Ruiz-Gaitán et al., 2018; Patterson et al., 2021). This silent reservoir poses a major challenge for infection control, as standard clinical cultures might not detect colonization unless active targeted surveillance screening (e.g., using axilla/groin composite swabs) is implemented, especially for high-risk patients or contacts of identified cases. The management of this patient reservoir, through early identification via surveillance and subsequent isolation precautions, is therefore the first critical step in breaking the chain of transmission.

### 4.2 Environmental contamination and persistence

The healthcare environment serves as a critical amplifier and long-term reservoir for *Candida auris*, directly enabling its sustained transmission within facilities (Rudramurthy et al., 2017; Kauser et al., 2022). A key determinant of its success as a nosocomial pathogen is its remarkable resilience on abiotic surfaces, significantly exceeding that of many other *Candida* species (Santana et al., 2023). Studies have demonstrated that *C. auris* can survive for extended periods, ranging from weeks to months, on a variety of common hospital surfaces such as plastic bed rails, stainless steel tables, curtains, and walls (Ware et al., 2025). This prolonged environmental persistence is compounded by its tolerance to common hospital disinfectants, including some quaternary ammonium compounds, allowing it to remain viable in inadequately cleaned areas. Contamination occurs continuously from colonized patients and through activities of healthcare workers. Consequently, high-touch surfaces near infected or colonized patients become heavily contaminated, creating a persistent source for cross-transmission (Alvarado-Socarras et al., 2021; Meletiadis et al., 2024). Effective interruption of this route relies on rigorous environmental cleaning and disinfection with agents proven effective against *C. auris*, such as hydrogen peroxide-based disinfectants or chlorine-based solutions, highlighting the environment’s role as a central target for infection prevention and control strategies (Ahmad and Asadzadeh, 2023; Kenters et al., 2019).

### 4.3 Healthcare workers as vectors

Healthcare workers (HCWs) act as the primary dynamic vectors for the transmission of *Candida auris* between the patient reservoir and the contaminated environment (Meletiadis et al., 2024; Mulet Bayona et al., 2020). Despite being transient, contamination of HCWs’ hands, gloves, and clothing during routine patient care activities—such as skin contact, handling of medical devices, or touching contaminated environmental surfaces—is a frequent and critical event. Without strict adherence to hand hygiene protocols (using alcohol-based hand rub or soap and water) and the consistent use of appropriate personal protective equipment (e.g., gloves and gowns), HCWs can inadvertently transfer *C. auris* from colonized or infected patients to susceptible patients, and from contaminated environments to clean areas (Mulet Bayona et al., 2020; Naicker et al., 2021). This role underscores the central importance of HCW behavior in either propagating or interrupting transmission cycles. Therefore, comprehensive education, ongoing training, and monitoring of compliance with infection control measures are essential to minimize the role of HCWs as vectors and to protect both patients and healthcare personnel from cross-transmission.

### 4.4 Medical devices as portals of entry

Contaminated medical devices constitute a high-risk portal of entry, directly facilitating invasive infections and amplifying the spread of *C. auris*. This risk is particularly acute for invasive devices such as central venous catheters, urinary catheters, and endotracheal tubes, which can bypass the body’s natural epithelial barriers, providing the fungus with direct access to the bloodstream or sterile sites (Amer et al., 2023; Parak et al., 2022). Equally concerning is the contamination of frequently used non-critical equipment like thermometers, blood pressure cuffs, and ultrasound probes, which serve as efficient vehicles for cross-transmission between patients (de Almeida et al., 2021). The threat is compounded by the remarkable ability of *C. auris* to form robust biofilms on both plastic and metallic surfaces. These biofilms confer enhanced resistance to antifungal agents and standard disinfection protocols, allowing the pathogen to persist on devices despite cleaning attempts. Consequently, stringent protocols for the cleaning, disinfection, and sterilization of reusable medical equipment, along with the use of single-patient disposable devices whenever possible, are critical measures to sever this direct transmission route.

## 5 Drug resistance and transmission of *Candida auris*

The drug resistance characteristics of *C. auris* present a significant challenge in its epidemiology and clinical treatment. Compared to other traditional *Candida* species, *C. auris* shows notable resistance to a variety of commonly used antifungal agents, especially fluconazole, amphotericin B, and other first-line antifungal drugs. This resistance not only complicates the clinical treatment of *C. auris* infections but also increases its potential for hospital transmission, thereby amplifying the risk of nosocomial infections (Mishra et al., 2023; Sanyaolu et al., 2022; Muñoz et al., 2018; Lockhart et al., 2023; Zhu et al., 2020). The drug resistance of *C. auris* is closely linked to its transmission, as the acquisition and spread of resistance enhance its continued proliferation within hospital environments. The following discusses the drug resistance features of *C. auris*, the impact of its resistance on transmission, and relevant prevention and control strategies.

### 5.1 Molecular mechanisms of antifungal resistance in *C. auris*

The multidrug resistance phenotype of *C. auris* is mediated by a complex interplay of several molecular mechanisms, including point mutations in target genes, overexpression of efflux pumps, and biofilm formation (Jacobs et al., 2022; Kim and Eom, 2021; Fatima et al., 2023). Understanding these mechanisms at the genetic level is crucial for developing diagnostic tools and overcoming treatment failures.

#### 5.1.1 Target-specific resistance mechanisms

*Candida auris* employs distinct molecular mechanisms to counteract the action of major antifungal drug classes. Resistance to azoles, particularly fluconazole, is the most prevalent (Barantsevich et al., 2020; Chen et al., 2018). This primarily occurs through two key strategies: firstly, missense mutations in the ERG11 gene (e.g., Y132F, K143R), which diminish the drug’s binding affinity to its target enzyme, lanosterol 14α-demethylase (Bing et al., 2024; Barantsevich et al., 2020); and secondly, the overexpression of efflux pumps (e.g., the ABC transporter Cdr1 and the MFS transporter Mdr1), which actively reduce intracellular drug concentration (Fan et al., 2021). The combination of these mechanisms frequently results in high-level, pan-azole resistance. Regarding echinocandins, which are crucial first-line agents, resistance is a significant concern and is predominantly driven by hot-spot mutations in the FKS1 gene (e.g., S639F) (Meletiadis et al., 2024). These mutations, affecting the catalytic subunit of *β*-(1,3)-D-glucan synthase, compromise drug efficacy and are strongly linked to clinical treatment failures. Finally, resistance to polyenes like amphotericin B, while less common, is often associated with mutations in other genes involved in ergosterol biosynthesis (e.g., ERG2, ERG3, ERG6), leading to alterations in the fungal cell membrane that reduce drug binding (Ahmad et al., 2019; Kenters et al., 2019; Ahmad and Asadzadeh, 2023).

#### 5.1.2 Broad-spectrum resistance mechanisms

Beyond drug-specific alterations, *C. auris* utilizes general strategies such as robust biofilm formation on both biotic (e.g., host tissues) and abiotic (e.g., medical devices) surfaces, which confers tolerance to multiple antifungal classes (Fatima et al., 2023; Dire et al., 2023). The biofilm matrix acts as a physical barrier that impedes drug penetration, while cells within biofilms often enter a metabolically dormant state, exhibiting enhanced antifungal tolerance and complicating the treatment of device-associated infections.

### 5.2 Acquisition and dissemination of resistance

The emergence and spread of resistance mechanisms in *Candida auris* are driven by several interconnected factors: genetic adaptation through *de novo* mutations under antifungal selective pressure enables strain-specific resistance development (Tian et al., 2024); horizontal gene transfer via mobile genetic elements (e.g., transposons) facilitates the rapid dissemination of resistance traits across strains (Chow et al., 2023); and persistent environmental selective pressure from widespread antifungal use in healthcare settings enriches resistant clones, particularly in vulnerable patient populations (Spruijtenburg et al., 2023).

### 5.3 The interplay between resistance and transmission

Drug resistance directly enhances the transmission potential of *C. auris* in healthcare environments. Resistant strains can persist for extended periods on contaminated surfaces (e.g., bed rails, walls) and medical equipment (e.g., ventilators, catheters) even in the presence of residual antifungal agents (Amer et al., 2023; Parak et al., 2022). This prolonged environmental survival, combined with cross-transmission via healthcare workers’ hands or contaminated devices, allows resistant clones to establish and sustain outbreaks in high-risk areas such as ICUs. Thus, resistance is not merely a treatment obstacle but a key factor fueling the epidemic spread of *C. auris*.

## 6 Prevention and control strategies

*Candida auris* has become a significant global public health challenge due to its multidrug resistance, widespread transmission in healthcare settings, and the severe infections it causes (Long et al., 2024; Du et al., 2020; Lone and Ahmad, 2019; Cristina et al., 2023). Therefore, effective prevention and control strategies for *C. auris* are crucial to reduce infection rates, control its transmission, and alleviate clinical treatment difficulties. This section discusses current prevention and control strategies for *C. auris*, focusing on infection control measures, early detection, rational use of antifungal drugs, and the development of new therapies.

### 6.1 Strict infection control measures

Since *C. auris* transmission mainly occurs in hospitals and healthcare facilities, particularly in high-risk environments such as ICUs, dialysis units, and operating rooms, infection control measures are especially important (Cristina et al., 2023; Lone and Ahmad, 2019; Long et al., 2024). Effective infection control can reduce the spread of *C. auris* and prevent the spread of resistant strains. First, hand hygiene and personal protective measures are the foundation for preventing *C. auris* transmission. Studies have shown that hand hygiene and disinfection significantly reduce the risk of spreading drug-resistant fungi in hospitals. Healthcare workers should strictly follow hand hygiene procedures after patient contact, handling medical devices, or interacting with patients. Additionally, wearing gloves, masks, and other personal protective equipment effectively prevents the spread of *C. auris* (Boyce, 2024; Jones et al., 2024).

Environmental cleaning and disinfection are equally crucial. *C. auris* can survive in hospital environments for extended periods, particularly on contaminated medical devices, bed linens, door handles, and other surfaces. Therefore, regular cleaning and disinfection, particularly in high-contact areas such as bedrails, bathrooms, and door handles, can effectively reduce the risk of transmission. Using disinfectants with effective antifungal agents can eliminate potential *C. auris* sources and reduce environmental contamination. Lastly, isolation and monitoring are key to preventing *C. auris* transmission. Once a case of *C. auris* infection is identified, immediate isolation measures are essential. Strict isolation should be implemented for immunocompromised patients, long-term hospitalized patients, and those with invasive medical devices to prevent cross-contamination. Strengthening infection control teams’ monitoring and tracking the epidemiology of *C. auris* can help quickly respond to outbreaks (Jones et al., 2024; Aldejohann et al., 2022; Wasylyshyn and Stoneman, 2024).

### 6.2 Early detection and diagnosis

Due to the potential harm and resistance of *C. auris*, early detection and diagnosis are critical for controlling infections. Timely and accurate detection allows for early intervention and isolation measures, preventing the infection from spreading. In recent years, molecular biology technologies, such as polymerase chain reaction (PCR), gene chips, and real-time quantitative PCR, have been widely used for early detection of *C. auris* (Crawford et al., 2022; Keighley et al., 2021). These technologies can quickly and sensitively detect specific *C. auris* genes, improving the accuracy of early diagnosis. In addition to molecular biology techniques, the traditional method of isolating and culturing *C. auris* from clinical samples is still used for diagnosis. Culturing allows for the acquisition of live cultures, which can then be used for further biological characterization, including budding morphology, biochemical reactions, and genetic identification (Kumar et al., 2017). Moreover, *C. auris*’s specific genotype can serve as a reference for its identification (Santana et al., 2024). Given the resistance issue, monitoring antifungal drug resistance is also crucial. Standardized drug susceptibility testing methods (such as agar dilution or E-test) can assist clinicians in choosing the optimal treatment and monitor changes in resistant strains.

### 6.3 Rational use of antifungal drugs

The rational use of antifungal drugs is another key strategy to prevent the expansion of *C. auris* resistance. Overuse or inappropriate use of antifungal drugs accelerates the development of drug resistance, making treatment more difficult (Munshi et al., 2024; Lone and Ahmad, 2019). Therefore, establishing appropriate antifungal drug use policies to ensure drug efficacy and safety is essential for controlling *C. auris*. Precision therapy is at the core of rational antifungal drug use. Clinicians should select the appropriate antifungal drugs based on drug susceptibility testing results. For example, for *C. auris* strains resistant to fluconazole or amphotericin B, doctors should prioritize alternative antifungal drugs such as voriconazole, itraconazole, or amorolfine, adjusting therapy based on the patient’s condition. Additionally, hospitals should establish guidelines for antifungal drug use to limit misuse and avoid unnecessary long-term antifungal treatment, particularly in cases where a diagnosis has not been confirmed, to reduce the selection pressure for resistant strains.

### 6.4 Development and exploration of new therapies

As the issue of *C. auris* resistance intensifies, traditional antifungal drugs are becoming less effective in treating the infection (Jacobs et al., 2022). To address this resistance problem, the development of new antifungal drugs and treatment strategies has become urgent. Current research is focused on the following areas: First, the development of new antifungal agents has become a key direction for researchers. These new drugs can not only overcome the resistance of *C. auris* but also provide more effective treatments by targeting its unique biological characteristics, such as the development of antifungal vaccines and targeted immunotherapy, which opens up new avenues for treatment (Akhtar et al., 2021; Singh et al., 2023). Second, considering that a single drug may not be sufficient to address resistant *C. auris* strains, combination therapies are emerging as a potential treatment strategy. By combining antifungal drugs with different mechanisms of action, treatment effectiveness can be enhanced, resistance can be reduced, and the success rate of treatment can be increased (Hernando-Ortiz et al., 2024).

## 7 Conclusion and future perspectives

The widespread transmission of *C. auris* globally, especially among immunocompromised patients and those with long-term hospital stays, has become a prominent public health issue (Lone and Ahmad, 2019). Its strong resistance to antifungal drugs and its adaptability in hospital environments make treatment exceptionally challenging. Traditional antifungal medications often fail to effectively control the infection, leading to increased patient mortality (Cristina et al., 2023; Long et al., 2024). Although progress has been made in antifungal drug management, hospital infection control measures, and early diagnostic technologies, the expansion of *C. auris* resistance and its survival ability in hospital settings still pose significant challenges for prevention and control (Mishra et al., 2023; Sanyaolu et al., 2022; Santana et al., 2023; Jacobs et al., 2022).

*C. auris* has become a major global public health threat due to its resistance, rapid transmission, and ability to survive for extended periods in healthcare environments. Although various prevention and control measures have been implemented, controlling *C. auris* transmission remains difficult due to the complexity of its resistance and transmission mechanisms (Mishra et al., 2023; Santana et al., 2023; Jacobs et al., 2022; Didik et al., 2023). Future research should focus on improving the accuracy of early diagnosis, optimizing antifungal treatment strategies, and exploring new prevention measures. Only through comprehensive and multifaceted control strategies can the spread of *C. auris* be effectively slowed, and the threat posed by its virulence be reduced.

Despite these challenges, the integrative transmission model proposed in this review (Table 1) highlights the interconnectedness of patients, the environment, devices, and healthcare workers in perpetuating *C. auris* outbreaks. Moving beyond a simple listing of factors, this model provides a practical roadmap for implementing targeted, evidence-based infection prevention and control bundles. Therefore, future research should not only focus on improving diagnostics and new drugs but also include validating the efficacy of each intervention strand within this model in diverse healthcare settings.

Future research should concentrate on several key areas: first, the development of new antifungal agents, particularly those capable of overcoming current resistant strains, such as therapies targeting *C. auris*’s unique virulence factors. Secondly, personalized treatment and precision medicine will enhance the efficiency of clinical treatments by optimizing therapeutic strategies based on drug susceptibility tests and patient-specific conditions. Additionally, diversified prevention strategies should continue to be strengthened. Infection control measures in hospitals, early detection technologies, and the education and training of healthcare workers should be continually improved to reduce the risk of *C. auris* transmission. Vaccine development and immunotherapy, as potential future treatments, also warrant attention. Study into *C. auris*’s immune evasion mechanisms could offer new strategies for preventing infection. Finally, global public health cooperation is essential. Only through strengthening international collaboration and information sharing, establishing global surveillance and emergency response systems, can we effectively address *C. auris*’s cross-border transmission and resistance issues. With these comprehensive strategies, we can better control the spread of *C. auris* and improve global capabilities in combating fungal infections.
